# Phytochemical Characterization of Two New Olive Oil Genotypes Growing in Southern Tunisia

**DOI:** 10.3390/molecules29173997

**Published:** 2024-08-23

**Authors:** Mbarka Ben Mohamed, Sihem Ben Ali, Gabriele Rocchetti, Samir Tlahig, Leila Bennani, Ferdaous Guasmi

**Affiliations:** 1 Dry Lands and Oases Cropping Laboratory, Institute of Arid Regions, El Fje, Medenine 4119, Tunisia; mbarka.bmohamed@yahoo.fr (M.B.M.); ben_ali.sihem@yahoo.fr (S.B.A.); samirtlahig@gmail.com (S.T.); mbleilon@live.fr (L.B.); guasmifer@yahoo.fr (F.G.); 2 Department of Animal Science, Food and Nutrition, Università Cattolica del Sacro Cuore, Via Emilia Parmense 84, 29122 Piacenza, Italy

**Keywords:** *Olea europaea* L., genotypes, olive oil, quality, phytochemical profile, antioxidant activity

## Abstract

This research can be considered as the first complete survey for the valorization of new olive genotypes cultivated in the South-East of Tunisia as well as their oils. The study aimed to characterize the phytochemical composition of virgin olive oil produced from two olive cultivars, namely Nourgou and Gousalani. The pomological characterization of fruits, the quality criteria and the phytochemical profile were quantified. Additionally, antioxidant activity was evaluated using Ferric reducing antioxidant power (FRAP) and Oxygen radical absorbance capacity (ORAC) tests to also obtain a bioactive characterization of these monovarietal olive oils. The obtained results revealed that the analyzed olive oils samples can be classified into Extra Virgin category (EVOO) according to the regulated physicochemical characteristics. Our findings showed a significant variability in the chemical parameters of the analyzed EVOO likely associated with the genetic potential, mainly for chlorophylls contents (1.37–1.64 mg/kg), in carotenoids pigments (3.97–10.86 mg/kg), in α-tocopherol (175.59–186.87 mg/kg), in sterols (1036.4–1931.4 mg/kg) in oleic acid (65.33–68.73%), in palmitic acid (C16:0) (13.32–17.48%), in linoleic acid (C18:2) (11.06–13.47%). Additionally, the HPLC-MS/MS analysis showed that the two EVOOs analyzed contained appreciable amounts of total polyphenols, ranging from 348.03 up to 516.16 mg/kg, in Nourgou and Gousalani oils, respectively. Regarding the individual phenolic compounds, the EVOO samples were mainly characterized by phenolic alcohols, phenolic acids, secoiridoids, verbascoside, flavonoids and phenolic aldehydes. The prevalent simple phenolics detected were secoiridoids with the dominance of the oleuropein aglycone in Gousalani oil. In addition, findings from in vitro antioxidant assays (FRAP and ORAC) revealed that the two studied oils possessed a powerful antiradical activity and a good reducing power capacity. In conclusion, these new EVOOs exhibited a superior quality compared to other Tunisian varieties, considering their antiradical activity and reducing power capacity.

## 1. Introduction

*Olea europaea* L. is a fruit tree species widely cultivated in the Mediterranean basin for its nutritional and healthy fruits. Virgin olive oil, which has outstanding nutritional, sensory, and functional properties [1], is an integral ingredient of the Mediterranean diet. This oil is beneficial for health due to its high levels of natural antioxidants, such as phenolic compounds and unsaturated fatty acids, which distinguish it from other vegetable oils. Consequently, interest in olive oil as a nutritional food source has developed outside the Mediterranean region, owing to its healthy virtues attributed to its rich chemical composition and abundance of other beneficial food components [2,3]. Olive oil can thus be considered an excellent source of natural bioactive compounds with significant antioxidant and positive effects on human health.

Recently, there has been an increasing global demand for olive oil due to its health benefits, including its impact on cancer, inflammation, diabetes, and cardiovascular and metabolic diseases. The antioxidant activities in olive oil are primarily related to health-promoting metabolites such as pigments, tocopherols, squalenes, sterols, and phenolic contents. The richness in these compounds makes olive oil one of the most important healthy edible oils worldwide.

In Tunisia, olive growing is one of the main agricultural and agri-food activities, playing a significant socioeconomic role. Tunisia, a southern Mediterranean region, is the fourth largest producer of olive oil, with a production of 200 thousand tons for the 2023–2024 season. It ranks first in olive oil production and export after the European Union, covering 30% of the total crop area with 1.96 million hectares of olive orchards. Tunisian olive growing is characterized by a rich varietal heritage [4], with olive plantations spread across all agricultural areas from north to south and east to west, constituting one of the important strategic sectors of Tunisia’s economy.

The major cultivated varieties in Tunisia are Chemlali in the south and center and Chetoui in the north, representing 56% and 12% of total olive tree surfaces, respectively [5]. In comparison, other Tunisian olive genotypes like Zalmati, Chemchali, Zarrazi, Gerboui, Toffehi, Fakhari, Tounsi, and Oueslati are considered minor varieties. These are grown in areas where farmers have selected the cultivars most adapted to the geographical conditions of the country. However, the yield and quantity of oil or olives produced from these secondary or rare varieties cannot be evaluated accurately because farmers often blend fruits from all olive varieties. According to the literature, most research has focused on the dominant varieties, with only a few studies on minor or secondary varieties. Consequently, to develop and enrich the olive sector in Tunisia, it is necessary to investigate and analyze these unstudied minor olive genotypes [6,7,8]. To the best of our knowledge, there is a lack of information on the phytochemical profiles of several unstudied rare varieties cultivated in different regions of southern Tunisia. Therefore, exploring olive tree varieties growing in these geographical sites is essential. This research can contribute to producing olive oils characterized by various sensory and chemical profiles, which could be recommended for large-scale plantations by Tunisian olive growers.

Nourgou and Gousalani, two new olive genotypes, were first discovered in a mountainous region in southeast Tunisia, Toujene (near Matmata-Gabes) [9]. The genotypic profiles of these new genotypes were generated using a robust set of SSR markers, resulting in their unambiguous identification and authentication compared to reference cultivars in three official collections: the germplasm ‘Boughrara’ Sfax, the World Cordoba olive germplasm bank, and the CREA-OFA collection. To the best of our knowledge, this is the first research article focusing on the chemical composition and antioxidant capacity of virgin olive oils obtained from these two rare Tunisian varieties, aiming to valorize them in terms of their bioactive profiles.

Investigating these unstudied *Olea europaea* cultivars and their oil profiles is crucial for gaining more information about rare Tunisian olive cultivars. Selecting new olive genotypes with high oil quality under arid and semi-arid Tunisian conditions can improve the olive oil production sector and diversify the country’s olive genetic resources. In this context, the aim of this study is to analyze the phytochemical profiles and in vitro antioxidant activities of two new olive oil genotypes, Nourgou and Gousalani.

## 2. Results and Discussion

### 2.1. Evaluation of the Pomological Characterization of Fruits

Pomological characteristics of the studied cultivars (fruit weight, maturity index, and fat content) are summarized in Table 1.

According to the results from ANOVA, the fruit weight (PMF) did not show any significant variation among the two olive genotypes analyzed. In contrast, a significant variation was observed in the other pomological characteristics (maturity index (MI) and fat content relative to dry weight) (*p* < 0.05).

#### 2.1.1. Fruit Weight

Table 1 revealed that the average fruit weight varies from 1.28 ± 0.02 (g) to 1.31 ± 0.03 (g), respectively, in the genotypes Nourgou and Gousalani. According to the classification proposed by [10], these varieties are classified very small, having a weight of olives not exceeding 2 g. The variation in average weight for different olive varieties can be explained by several factors, such as the geographical area and the hydrous conditions [11].

The average fruit weight is a highly sought-after agronomic characteristic. Its values can be used to estimate the productivity potential of the specific variety and reflect its yield and suitability for multiple uses [12,13,14].

#### 2.1.2. Maturity Index (MI)

Olives were collected at the optimal harvest period (November–December). According to [15], during this period, the olive tree is characterized by optimal maturity and a high oil yield. In Table 1, it is highlighted that the maturity index is estimated at 2.19 ± 0.111 and 3.50 ± 0.556 in the Gousalani and Nourgou, respectively. Our findings were in accordance with [16], who reported that the optimal ripening index is an MI equal to 3 for some Tunisian varieties like Zalmati, Chemchali, Zarrazi, and Chemlali Zarzis, and for the Chemlali Sfax cultivar, the MI is between 2.5 and 3. According to [17], the olive oils obtained from olives harvested in a ripening index between 2.5 and 3.5 were characterized by an excellent chemical composition and good sensory quality.

#### 2.1.3. Fat Content Relative to Dry Weight

According to [18], this parameter does not constitute a criterion for determining the quality of the oil but makes it possible to determine the optimal harvest date. Oil content from an olive genotype is the main tool for determining its acceptability among olive growers and processors [14]. Table 1 shows that the olive oil yield for the two EVOOs is important, ranging on average from 47% for the Gousalani to 38.41% for the Nourgou cultivar. In a previous research, obtained results have shown that every variety has a variation in oil recovery, which is generally due to its genetic profile, in addition to some external parameters such as climate, temperature, and soil, which have a significant impact; however, temperature is considered the most influential factor in oil quantity [19]. Ref. [20] also reported a positive correlation between oil content variation and olives size that was affected by exogenous and endogenous parameters. In the same way, ref. [21] revealed that the highest olive size, pit weight, and fatty acid composition directly correlate with oil content percentage.

### 2.2. Quality Criteria

Table 2 illustrates the physicochemical characteristics of the extracted olive oils. Overall, we found acidity values ≤ 0.8%, a K232 ≤ 2.5, followed by a K270 ≤ 0.22, and a peroxide value ≤ 20 meq O_2_/kg.

Therefore, oils from Nourgou and Gousalani could be classified as extra virgin olive oil (EVOO) in agreement with the standard limits described by [22] for the highest quality extra virgin olive oil category. In this study, necessary precautions (fresh and healthy fruits, optimal maturation period, and immediate extraction) were taken into consideration to obtain good-quality extracted oils.

Our findings align with those reported in the literature for other Tunisian virgin olive oils [7,23]. These quality criteria are influenced by factors affecting both the olives, such as olive fly attacks, harvesting methods, and conditions of transport and storage; and the oil, including processing technologies and storage conditions [24,25].

### 2.3. Pigment Contents

Chlorophyll and carotenoid pigments were determined using colorimetric methods. The results from ANOVA showed that the effect of genotype factor on pigment contents was highly significant (*p* < 0.001) (Table 3).

For the analyzed EVOO samples, chlorophyll contents were found at an average quantity between 1.37 and 1.64 mg/kg, respectively, for the Nourgou and Gousalani cultivars. Regarding the carotenoid contents, the highest amount was found in the Nourgou (10.86 mg/kg), while the lowest level (3.97 mg/kg) was recorded in the Gousalani cultivar. These findings agreed with those of previous research concerning other varieties grown in Tunisia [26].

The determination of coloring pigments is not recommended for an olive oil marketing standard. In addition, color is a basic attribute to determine olive oil characteristics; consequently, many consumers associate it with the concept of quality. The color of olive oil is directly correlated to the pigment contents as a characterizing factor and quality parameter [27]. They are a natural antioxidant involved in the phenomenon of oil oxidation; their presence in sufficient concentration in the oil makes it possible to delay the oxidation and to preserve the quality criteria during the storage period. According to several researchers [28,29,30], these olive compounds are potentially affected by various factors, such as olive genotype, pedoclimatic conditions, olives ripening, and processing technology.

### 2.4. Tocopherol Content

Tocopherols are the main group of primary antioxidants occurring in vegetable oils and fats. α-tocopherol is considered the major fraction in virgin olive oil, representing nearly 95% of the total tocopherols content, while γ-tocopherol is present in low concentrations (<10%), and β-tocopherol exists in very low content in the form of traces [31]. The results from ANOVA (Table 3) showed that the effect of genotype factor on α-tocopherol content was statistically highly significant (*p* < 0.001).

Among the EVOOs analyzed, the higher concentration of α-tocopherol was recorded in oil obtained from the Nourgou cultivar (186.866 ± 1.093 mg/kg), and the lower quantity was observed in oil extracted from the Gousalani cultivar (175.593 ± 1.011 mg/kg). These quantities agreed with those reported for good-quality oils, which range from 100 to 300 mg/kg [16]. Our results were low compared to the corresponding values of olive oil samples from Tunisia [32] (485.1–765.3 mg/kg). On the other hand, they are comparable for the results found by [7]. Ref. [33] mentioned that Tunisian olive oils are very rich in tocopherols compared to European oils. This is confirmed by the results found by [34], who revealed that oils of Tunisian origin are the richest in α-tocopherol compared to other oils analyzed from Spain and Morocco. Several authors have mentioned that tocopherol contents were highly variety-dependent, and these differences were probably attributed to genotype characteristics and the metabolic nature of each variety [35,36].

### 2.5. Fatty Acid Composition

Fatty acid composition is considered an essential characteristic for olive oil: firstly, for the detection of adulteration and fraud; secondly, it has previously been used as a criterion for classifying olive oils [37,38]. Table 4 shows the methyl ester fatty acid composition in the two studied olive oil samples.

Qualitatively, the analyzed olive oils present a similar fatty acids profile; in contrast, quantitatively, differences were recorded. Overall, 11 fatty acids have been detected in the EVOOs in this study. Oleic acid (C18:1) is the most abundant, followed by linoleic acid (C18:2), palmitic acid (C16:0), stearic acid (C18:0), and linolenic acid (C18:3). This finding agrees with results reported by [23], who showed that oleic acid (C18:1), linoleic acid (C18:2), and palmitic acid (C16:0) are the major fatty acids in olive oil and vary significantly depending on the cultivar genotype. These fatty acids are very important criteria for determining the quality and authenticity of olive oil. The results recorded in Table 4 revealed that in the two analyzed EVOOs, the amount of oleic acid represents more than 55%, the palmitic acid quantity was less than 20%, and the linoleic acid level did not exceed 21%. Oleic acid is a principal monounsaturated fatty acid; the highest value was observed in EVOO from the Gousalani (68.73 ± 0.651%), and the lowest percentage was recorded in EVOO from Nourgou (65.33 ± 0.588%). On the other hand, the latter is characterized by the highest content of the major saturated fatty acid (C16:0) (17.48 ± 0.50%), and the lowest percentage is recorded in olive oil produced from the Gousalani cultivar (13.32 ± 0.29%). Concerning the stearic acid, another main saturated acid, the amounts obtained are comparable for the two analyzed EVOOs. Linoleic acid (C18:2) is an important fatty acid for human nutrition. It is the first polyunsaturated fatty acid in olive oil correlated negatively to oxidative stability. Other fatty acids are considered minor. The determination of these minor fatty acids is very necessary for the characterization of olive varieties [39] and for the precise knowledge of lipid profiles during the authentication of the varietal origin of olive oils [40]. According to the results from ANOVA, the three fatty acids C14:0, C17:0, and C17:1 did not show any significant variation among the EVOO samples analyzed. In contrast, a significant variation was observed in the level of C18:0 (*p* < 0.05). The concentration of C18:1 and C18:3 revealed a very significant difference (*p* < 0.01), and highly significant variation (*p* < 0.001) was recorded in the other detected fatty acids (C16:0, C16:1, C18:2, C20:0, C20:1). This difference is mainly explained by the genetic factor.

### 2.6. Sterolic Content

Sterols constitute an important component; they are mainly found in all fats and oils, and they are also indicative of the purity of vegetable oils. The sterolic amounts of olive oil varied from 1000 up to 3000 mg/kg [41]. These contents vary depending on different factors, including genetic factor, fruit ripening period, geographical origin of olives, climate, and irrigation system [42,43].

Table 5 exhibited the total sterols content, estimated as cholesterol equivalents, of the two EVOOs analyzed. It was around 1000 mg/kg in both cultivars, which is within the limit recommended by [44] for the “extra virgin” olive oil category. Consequently, these oils represent a good source of health-promoting compounds.

Table 5 showed that the EVOO produced from Gousalani genotype had a significantly higher sterolic content (1931.4 ± 251.4 mg/kg cholesterol equivalents) compared to Nourgou genotype (1036.4 ± 147.8 mg/kg cholesterol equivalents).

Total sterol content from Nourgou and Gousalani are in concordance with other Tunisian olive oil varieties described in the scientific literature [45,46,47]. These authors reported that the concentration of total sterols in monovarietal Tunisian oils ranged between 1082 and 2291 mg/kg of total sterols. In addition, sterol content can be used to determine olive oil adulteration. In addition, it has been recently suggested to be used in virgin olive oils classification [45] due to its benefits [48].

### 2.7. Phenolic Composition

The EVOO samples analyzed present a high amount of phenolic substances, as shown in Table 5, where the cumulative concentration of individual phenolic content is evaluated as a function of genotype cultivar. The results from ANOVA showed that the effect of genotype factor on phenolic composition was statistically highly significant (*p* < 0.001). The olive oil extracted from the Gousalani cultivar showed a higher value in total phenols (516.16 mg/kg) than the Nourgou oil (348.03 mg/kg).

Our data agree with those mentioned in previous studies, which showed that olive oil produced from different Tunisian varieties could be considered a potential source of phenolic compounds [8,49]. According to several authors, the content of phenolic compounds is highly dependent from the cultivar under consideration [6,50]. Ref. [51] reported that genetic factors have an impact on olive oil quality, mainly its phenolic profile.

Table 6 shows that 19 compounds were recorded in the two olive oils analyzed. A remarkable difference was recorded for the different phenolic substances depending on olive oil genotype. In this regard, ref. [52] reported that there is a difference in both qualitative and quantitative profiles of phenolic constituents from the analyzed olive fruit samples collected from various areas.

EVOO samples were characterized by appreciable concentrations of phenolic alcohols, phenolic acids, secoiridoids, verbascoside, flavonoids, and phenolic aldehydes. The common simple phenols detected in the olive oils analyzed were secoiridoids (i.e., oleuropein aglycone, oleuropein, and ligstroside aglycone). These findings were in accordance with the results of [53], who revealed that secoiridoids, oleuropein derivatives, are the most predominant among phenolic antioxidants of extra virgin olive oils.

EVOO extracted from the Gousalani cultivar was characterized by higher values than the Nourgou cultivar. In fact, oleuropein aglycone (164.99 mg/kg) dominated the three others secoiridoids. Several authors have reported that secoiridoids were the most abundant phenolic fraction in olive oils produced from some Tunisian varieties: Sehli, Baldi, Chétoui, Chemchali, Besbessi, Touffehi, Zalmati, Jemri, Neb Jmel, Tounsi, and Fakhari [32,54,55]. The second major phenolic group detected was phenolic alcohols, mainly hydroxytyrosol and tyrosol. According to [30,56], they were derived from hydrolysis of oleuropein aglycone and ligstroside aglycone. The value of these two phenolic compounds is very important, as these antioxidant substances contribute to the prevention of blood lipids from oxidative stress [44].

The content of hydroxytyrosol of analyzed EVOO varied from 2.56 to 6.19 mg/kg for the Nourgou and Gousalani genotypes, respectively. On the other hand, a high value of tyrosol is recorded in the Nourgou oil (7.14 mg/kg), and a low value is observed in the Gousalani oil (4.77 mg/kg). The phenolic alcohols present in the studied EVOO were high compared to the corresponding values for olive oil samples analyzed by [23].

In addition to these phenolic components, phenolic acids (e.g., caffeic, vanillic, *p*-coumaric, ferulic, and *o*-coumaric) were also detected in the analyzed oils. Gousalani olive oil was characterized by a high content of *o-* coumaric acid (12.60 mg/kg), and the olive oil produced from the Nourgou cultivar presents high amounts of vanillic acid (1.46 mg/kg) and *p*-coumaric acid (2.53 mg/kg).

The other identified phenolic acids were observed in low quantities. These findings were also confirmed by [57], who admitted that phenolic acids were recorded as minor metabolites in olive oil. Considering the phenolic profile, flavonoids were the most frequent class of phenols, with seven compounds detected and quantified: catechin, luteolin-7-*O*-glucoside, rutin, luteolin-4-*O*-glucoside, luteolin, apigenin, and diosmetin.

The observed major constituent of the flavonoids group was luteolin-4-*O*-glucoside. Its content ranged from 4.53 mg/kg in Nourgou oil to 26.83 mg/kg in Gousalani oil. Regarding the other detected phenolic compounds, they were characterized by variable concentrations, likely depending on genetic factors. In addition to these phenolic compounds, other phenols were detected in EVOO samples analyzed in this study, such as vanillin, which was present in very low quantities in both oils under investigation (varied from 0.47 to 0.83 mg/kg). Verbascoside was also detected in the olive oils studied. It was present in considerable amount; the highest content was found in Nourgou oil (7.00 mg/kg), and the lowest value was noted in Gousalani oil (4.63 mg/kg).

Overall, statistical analysis revealed a significant variation of the majority of individual phenolic compounds, which depended mainly on the genotype (*p* < 0.001), except for catechin and diosmetin (Table 6).

**Figure 1:**
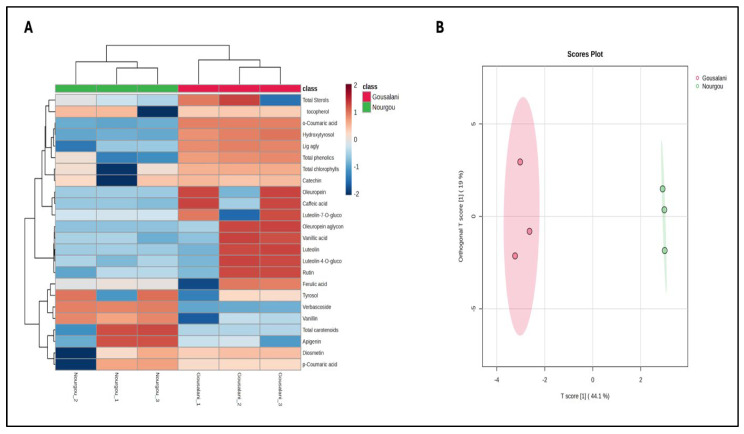
Unsupervised hierarchical clustering analysis (**A**) and orthogonal projections to latent structures discriminant analysis (**B**) plots. VIP [t] = prediction ability between-groups; VIP [ortho-t] = prediction ability within-groups.

A multivariate statistical approach was used to better discriminate the two EVOO samples. The results of both unsupervised and supervised investigations are reported as Figure 1A (heat map from unsupervised hierarchical clustering analysis) and Figure 1B (score plot from OPLS-DA prediction modelling). Overall, Figure 1 clearly demonstrates a huge diversity in terms of the phytochemical fingerprint when comparing the two genotypes; in particular, the majority of phytochemicals were better represented in the Gousalani oil sample (Figure 1A). Interestingly, the OPLS-DA prediction model showed more than acceptable model parameters related to both goodness of fitting and prediction (both higher than 90%), thus confirming the ability of the annotated phytochemicals to discriminate the two EVOO samples.

Finally, the best phytochemicals in terms of cultivar discrimination were extrapolated through a VIP approach, considering as a minimum cut-off a VIP score > 1. The results are reported in Table 7. Interestingly, we found 10 features mostly associated with cultivar discrimination, with o-coumaric acid, verbascoside, and hydroxytyrosol possessing the highest prediction abilities (Table 7), exclusively related with their between-group separation ability. On the other hand, three additional phenolics were particularly able to also explain a within-group separation, namely oleuropein aglycone, vanillic acid, and vanillin (Table 7).

Previous works [58] have affirmed that the development of metabolomic profiling approaches for phenolic fingerprints could enhance the understanding of the biological and nutritional properties of olive oil, as well as promote their use for traceability and authenticity purposes. Furthermore, according to [59], in addition to their impacts on food quality, the application of untargeted metabolomics profiling of sterols and polyphenols coupled to multivariate chemometrics appears to be a very promising tool to discriminate different EVOO samples of different geographical origins (Tunisian vs. Italian). The amount of phenolic compounds in olive leaves, fruits, and oil is not stable and varies widely [60]. The content of polyphenols and oil in olive drupes varied significantly according to many factors such as cultivar or variety genotype [61], plant individuals and population [60], the region of its production, environmental and pedoclimatic conditions, agricultural practices, ripening stage of olives, and the fruit processing method [62,63], together with extraction methods, extraction solvents [64], and the systems used to separate oil from olive pastes. The conditions of storage are also important.

### 2.8. In Vitro Antioxidant Activity Characterization

The antioxidant characteristic should not be evaluated in vitro by means of a single antioxidant assay model, due mostly to the huge chemical variability of antioxidant metabolites together with the complexity of the food matrix [65]. In our study, the antioxidant potential was assessed as both reducing power and radical scavenging by using FRAP and ORAC methods, respectively.

An examination of Table 8 shows that the results from ANOVA revealed that the effect of genotype factor on antioxidant activity was very significant (*p* < 0.01) with the FRAP assay and highly significant (*p* < 0.001) with the ORAC method.

The antioxidant capacity evaluated by FRAP test ranged from 15.66 to 50.00 mg gallic acid equivalent/100 g for the Nourgou and Gousalani olive oils, respectively. Regarding the ORAC radical scavenging capacity, different results were observed between the two EVOO samples analyzed. The highest antioxidant potential was recorded in the Gousalani oil extract (8796 mM TEAC), whereas the lowest activity was shown in the Nourgou oil extract (2024.5 mM TEAC).

Based on the classification proposed by [66], which suggested four categories of ORAC quality, these two olive oils analyzed in this study can be characterized by a top quality of ORAC activity. The ranges were the following: 1–4: low-quality EVOO; 4–8: intermediate-quality EVOO; 8–12: high-quality EVOO; >12: top-quality EVOO.

FRAP and ORAC tests revealed that the two EVOOs are characterized by powerful antiradical activity and good reducing power capacity. Our results agreed with several studies that have proven that olive oil is characterized by a significant antioxidant activity. This is comparable to reference antioxidants such as BHT, as measured by various methods like the DPPH test, FRAP test, ABTS test, and β-carotene bleaching test [67,68]. According to [69], leaves, pulp, and stone extracts from Tunisian olive cultivars ‘Zarrazi’ and ‘Chemlali’, cultivated in situ, and two oleaster trees from the natural ecosystem in southern Tunisia showed high antioxidant capacity according to DPPH and ABTS assays. Equally, ref. [70] reported that the Tunisian Chetoui variety had an important antioxidant property (which varied between extracts from different organs), as shown by various methods such as DPPH, FRAP, ORAC, and β-carotene-linoleic acid bleaching assays. Ref. [71] reported that a Tunisian commercial olive oil shows very high antioxidant inhibition activity (84.96%) in comparison with olive oils produced from various Algerian regions. Virgin olive oil is a promising source of bioactive and natural antioxidants that can proceed through different mechanisms to confer an effective defense system against free radical attack [72]. According to [73], olive oil contains bioactive compounds (with strong antioxidant potential). It was clear that EVOOs are antioxidant in nature and can be used as a healthy substitute in place of other cooking oils.

### 2.9. Correlation Analyses

Olive oil is one of the edible oils with a high price in the fats and oils industry. Several antioxidant assays, in vitro and in vivo, revealed that olive oils are good sources of antioxidants. A correlation analyses among the various parameters was investigated especially to analyze how the differences revealed by antioxidant capacity values estimated by the two methods can be explained on the basis of the concentrations of the phytochemical compounds, or in particular which group of metabolites were responsible for the antioxidant property variability. The results of the correlation are of great importance to determine if the antioxidant activity was controlled by several direct and indirect metabolites.

**Figure 2:**
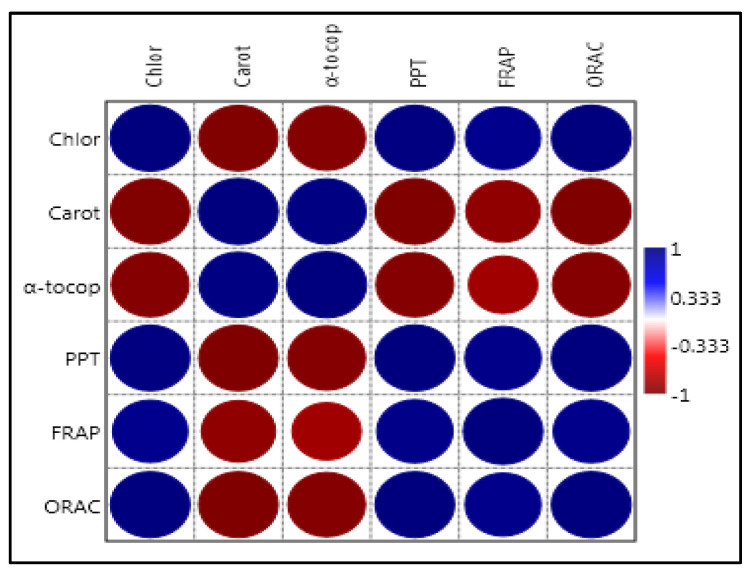
Correlation coefficient plot considering the different parameters evaluated in this work.

The results obtained showed a considerable relationship between several molecules detected and the antioxidant capacity evaluated. Estimates of the correlation are shown in Figure 2.

The correlation coefficients (Figure 2) of each compound revealed the presence of significant and positive correlation within the two methods, FRAP and ORAC. A positive and significant correlation was observed between the chlorophyll pigments; the total phenols; the total sterols; the fatty acids (mainly (C16:0), (C16:1), (C17:0), (C18:0), and (C18:3)); the phenolic alcohols (hydroxytyrosol), the secoiridoids (oleuropein aglycone, oleuropein, ligstroside aglycone), the phenolic acids (caffeic, ferulic, *o*-coumaric), the flavonoids (rutin, luteolin-4-*O*-glucoside, luteolin), and the antioxidant activity with the two methods. High correlation coefficients were found, with R values higher than 0.6, ranging from 0.81222 to 0.963 for the FRAP assay and from 0.89548 to 0.99833 for the ORAC test.

These results agree with those reported by [74], which stated that the antioxidant activities are mainly correlated to the health-promoting substances of olive oil, such as phenolic components, pigments, squalene, tocopherols, and sterols. These bioactive metabolites make olive oil one of the most important healthy edible oils worldwide. In the same context, ref. [75] revealed a positive correlation between the total phenol and flavonoids content and DPPH radical-scavenging activity; they reported that this finding could be ascribed to the highest contents of the total phenols and flavonoids, particularly ortho-hydroxylated phenolics, such as hydroxytyrosol and oleuropein aglycone. According to [76], a linear positive relationship between the total phenols content and antioxidant activity was observed, and the higher contents of total phenols in Tunisian oil were positively correlated with antioxidant activities in comparison with the other olive oils analyzed. The correlation analysis carried out on the different phenolic classes revealed that the total phenolic, flavonoid, and ortho-di-phenolic content were correlated with radical scavenging activity [77]. It has been demonstrated that bioactive phenolic compounds of olive oil are mainly responsible for its antioxidant or free-radical-scavenging activity [78].

## 3. Materials and Methods

### 3.1. Area of Study and Plant Material

Olive samples from Gousalani and Nourgou cultivars were collected from a mountainous area located in the region of Toujene (near to Matmata, in the government of Gabes). This region is situated in the south-east of Tunisia (latitude: 33.46; longitude: 10.13; altitude: 453 m), and it is characterized by an arid to semi-arid climate. Olive trees were the same age (30–40 years old), planted in the same pedoclimatic conditions, and under rain-fed conditions without fertilization and without pesticide application. For oil production, three fruit samples were taken during the same harvest period (November–December) from each olive genotype. Olives were collected and cleaned manually of all leaves and branches.

### 3.2. Pomological Characterization of Fruits

The average fruit weight (g) was calculated systematically from 100 fresh fruits randomly selected from the entire canopy of the tree from all directions. The fat content relative to dry weight parameter was determined by nuclear magnetic resonance (NMR) technique using an Oxford 4000 spectrometer (Oxford Instruments, Abingdon, Oxfordshire, UK), according to [79]. The maturity index (MI) was estimated according to the method described by [80], based on visual appreciation of the color samples of 100 fruits according to a color scale varying from 0 (skin color green-intense) to 7 (black peel color and all purple pulp).

### 3.3. Preparation of Virgin Olive Oil Samples

After harvesting, the olives were washed, and leaves were removed. Oil extraction was carried out using a laboratory extraction system called an oleodoseur (composed of crusher, vertical malaxator, and centrifuge) from handpicked fresh fruits at maturity time (2.5 to 3 kg), without storage time before extraction. Afterwards, natural decantations of the obtained oils were filled in dark glass bottles and stored at 4 °C until analysis.

### 3.4. Calculation of Quality Criteria

Estimation of free acidity, peroxide value (IP), and UV spectrophotometric indices (K232 and K270) were evaluated according to the official methods described by [81,82,83].

### 3.5. Determination of Chlorophyll and Carotenoid Contents

Chlorophyll determination was analyzed following the method described by [84], based on spectrophotometric quantification through detecting the absorbances at 630, 670, and 710 nm. Virgin olive oil samples were filled directly into a 1 cm pathlength glass cell (L), and the pure carbon tetrachloride was utilized as a control.

The chlorophyll compound was estimated using the following formula:

Chlorophyll (mg/kg) = (A670 − (A630 + A710)/2)/(0.1086 × L)

The carotenoid fraction was determined from the absorption spectra at 470 nm of 3 g of olive oil dissolved in 25 mL of cyclohexane according to the following method [85]:

Carotene (mg/kg) = (A470 × 25 × 1000)/(E × 75)

With E, specific extinction is equal to 2000.

### 3.6. Screening of Lipid Profile

#### 3.6.1. Determination of α-tocopherol Content 

The α-tocopherol content was estimated in virgin olive oils samples according to the previously published method reported in [86]. First, 1 g of extracted oil was dissolved in 10 mL of hexane, and then 20 μL of the solution was hand-injected into the HPLC (JASCO, Tokyo, Japan) on a LiChrospher Si (250 mm × 4.6 mm, particle size 5 μm) column. α-Tocopherol separation was achieved with an isocratic elution of hexane/isopropanol (99.5:0.5; *v*/*v*) at a flow rate of 1 mL/min. The fluorescence detector was set at 290 nm excitation wavelength and 330 nm emission wavelength. The identification and quantification of chromatographic peak were made by comparison to the response of α-tocopherol standard (Sigma-Aldrich, Co., St. Louis, MO, USA).

#### 3.6.2. Evaluation of the Fatty Acid Profile

In order to determine fatty acid composition, the methyl esters were extracted from olive oils and analyzed by gas chromatography (GC-MS: QP2010 ULTRA/SHI-MADZU/SUPEL, COWAXTM 10/FUSED SILICA capillary column 30 m × 0.25 µm film thickness) after cold saponification by mixing a solution of 0.2 g of oil and 3 mL of hexane with 0.4 mL of 2N methanolic potassium hydroxide, following the analytical methods established by [81]. Identification of fatty acid was obtained by comparing their retention times with those of standard compounds, and results were expressed as percent (%) of relative area.

#### 3.6.3. Determination of Total Sterols

The total sterolic contents of EVOO methanol extracts were gained as previously described by [87], with small modifications. Total sterols were extracted in triplicate from each sample as follows: an aliquot (3 g) of oil was accurately weighed into a conical centrifuge tube and added with 3 mL of 80% methanol solution (*v*/*v*) (LCMS grade, VWR, Milan Italy). The mixtures were vortexed vigorously and then centrifuged at 6000× *g* for 10 min at 4 °C. The methanol fractions were collected, while the residues were rejected. The resulting supernatants were filtered through 0.22 μm cellulose syringe filters and stored in amber vials at −20 °C until the following analysis through liquid chromatography mass spectrometry.

The sterolic fingerprints of EVOO methanol extracts were gained as previously described by [87], with small modifications. Analysis was carried out through ultra-high-performance liquid chromatography coupled to quadrupole-time-of-flight mass spectrometry by an electrospray ionization source (UHPLC-ESI/QTOF). A 1290 liquid chromatography system, equipped with a binary pump and a Dual Electrospray JetStream ionization source and coupled to a G6550 mass spectrometer detector (all from Agilent Technologies, Santa Clara, CA, USA), was used. The mass spectrometer was operating in positive ionization mode to acquire accurate masses in the 50–1000 *m*/*z* range. The chromatographic separation was performed on an Agilent Zorbax eclipse plus C18 analytical column (50 × 2.1 mm, 1.8 μm) and water–methanol gradient elution (from 10% to 90% organic in 34 min). The injection volume was 3 μL per sample. Source conditions were as follows: nitrogen was used both as sheath gas (10 L/min and 350 °C) and as drying gas (8 L/min and 330 °C), nebulizer pressure was 60 psig, nozzle voltage was 300 V, and capillary voltage was 3.5 kV. The raw data processing was carried out using the software Profinder B.07 (Agilent Technologies, Santa Clara, CA, USA), based on the “find-by-formula” algorithm. Compound identification was recursively carried out considering both accurate mass and isotopic pattern (isotope spacing and isotope ratio). A custom database, LIPID MAPS (Lipidomics Gateway, http://www.lipidmaps.org/ (accessed on 1 March 2024)), was used as a reference for identification, adopting 5 ppm tolerance for mass accuracy. Data pre-processing (mass and retention time alignment, compounds filtering) was realized in the software Profinder B.07, and only those compounds identified within 100% of replications in at least one treatment were retained. This processed dataset was finally used for statistics and chemometrics. A calibration curve of cholesterol (Sigma grade, Sigma-Aldrich, St. Louis, MO, USA) was used to estimate the total sterols content.

### 3.7. Determination of Phenolic Compounds

#### 3.7.1. Extraction Step

Phenolic compounds were obtained by liquid–liquid extraction using HPLC. Their separation was carried out with the same extraction conditions reported by [88]. Briefly, 3 g of the oil sample was added to 3 mL of n-hexane and 5 mL of a methanol/water (80:20, *v*/*v*) solution in a 20 mL centrifuge tube. After vigorous mixing for 10 min, it was extracted in an ultrasonic bath at room temperature for 15 min. They were centrifuged for 25 min at 5000 rpm/min. The supernatant phase obtained was filtered through a 0.45 µm polyvinylidene fluoride (PVDF) syringe filter into a vial and injected into the LC-MS/MS system.

#### 3.7.2. LC/MS Analysis

The analyses of filtered extra virgin olive oil (EVOO) extracts were conducted using a Sciex Applied Biosystem API 4000 Q-Trap mass spectrometric system. The instrument was run in negative ion mode using multiple reactions monitoring (MRM) tandem mass spectrometric acquisitions. The ion spray voltage (IS) was 4500 V, the curtain gas was 20 psi, the temperature was 400 °C, and the ion source gas pressures were 35 and 45 psi. Individual collision gas thickness (CAD) medium, entrance potential (EP), declustering potential (DP), entrance collision energy (CE), and exit collision energy (CXP) were optimized for each MRM transition. The separation was realized using an Eclipse XDB-C8-A HPLC column (5 µm particle size, 50 mm length, and 4.6 mm i.d. from Agilent Technologies, Santa Clara, CA, USA). The injection volume was 10 µL with a mobile phase flow rate of 350 µL/min. The binary mobile phase was made up of 0.1% aqueous formic acid and methanol gradient (the methanol was increased from 10% to 100% in 20 min). Peak assignment relative to the different compounds detected could be identified by comparing their retention time with those of pure standards and by MRM transitions. Reference standards were purchased from Extra synthesis (GenayCedex, Genay, France) and Sigma-Aldrich (Riedel-de Haen, Laborchemikalien, Seelze, Germany). Methanol and formic acid were LC/MS grade and bought from VWR International. The aqueous solutions were prepared using ultrapure water, with a resistivity of 18.2 MΩ cm, provided by a Milli-Q Plus system (Millipore, Bedford, MA, USA).

### 3.8. In Vitro Antioxidant Activity

The antioxidant capacity of EVOO extracts was determined by means of two different methods, measuring the ferric reducing antioxidant power (FRAP) and the oxygen radical absorbance capacity (ORAC). The FRAP and ORAC assays were evaluated using a Synergy HT Multi-Detection Microplate Reader (BioTek instruments Ibc., Winooski, VT, USA) following the method performed by [89], with some modifications.

### 3.9. Statistical Analysis

All experimental assays were conducted in three replications (n = 3), and the data expressed as means ± standard deviation. Data were analyzed for statistical significance by one-way analysis of variance (ANOVA), and significant differences at a 5% level were estimated using Student’s *t*-test. The software XLSTAT 2019.2.2.59614 was used to perform these analyses. Correlation analyses were evaluated following the Pearson’s correlation coefficient method using corrplot (V0.92, 2021) packages in R. Finally, the different datasets were elaborated through the software MetaboAnalyst 6.0 for both unsupervised and supervised statistical analysis, in order to extrapolate the best features for prediction ability between the two cultivars.

## 4. Conclusions

As a first study, the phytochemical characterization and antioxidant activity analysis of two new olive oil genotypes, namely Gousalani and Nourgou, provided interesting results. A significant variability was revealed in the chemical parameters of analyzed EVOO, mainly for chlorophyll content, carotenoid pigments, α-tocopherol, total sterols, oleic acid, palmitic acid, linoleic acid, and total polyphenols. The majority of individual phenolic compounds detected in the EVOO samples showed a significant variation mainly dependent on the genotype factor. Additionally, a chemometric multivariate statistical approach was applied to the measured phytochemicals, which was considered a robust tool to discriminate the two EVOO samples. New EVOOs exhibited a superior quality compared to other Tunisian varieties, with a strong antiradical activity and reducing power, as demonstrated by FRAP and ORAC tests. This research supports the potential for marketing Nourgou and Gousalani monovarietal oils due to their excellent quality, chemical composition, phenolic content, and antioxidant activity. This study serves as a starting point for promoting these varieties among Tunisian farmers for future large-scale cultivation.

## Figures and Tables

**Table 1:** The pomological characteristics of different samples studied.

Cultivars	Fruit Weight (PMF) (g)	Maturity Index (MI)	Fat Content Relative to Dry Weight
Nourgou	1.28 ± 0.02 ^a^	3.50 ± 0.556 ^a^	38.41 ± 0.078 ^b^
Gousalani	1.31 ± 0.03 ^a^	2.19 ± 0.111 ^b^	47.00 ± 0.158 ^a^

The data are expressed as mean values ± standard deviation; different letters indicate homogenous sub-groups from ANOVA (post hoc, *p* < 0.05).

**Table 2:** Quality criteria of the two studied EVOO.

Cultivar	Acidity	K232	K270	Peroxide Value
Nourgou	0.39 ± 0.026 ^b^	2.29 ± 0.036 ^a^	0.17 ± 0.013 ^a^	1.09 ± 0.017 ^a^
Gousalani	0.49 ± 0.045 ^a^	2.22 ± 0.036 ^a^	0.19 ± 0.01 ^a^	1.12 ± 0.017 ^a^

The data are presented as mean values ± standard deviation (n = 3). Different lowercase (^a,b^) letters denote significant differences according to Student’s *t*-test (*p* < 0.05).

**Table 3:** Total chlorophylls, carotenoids, and alpha-tocopherol (mg/kg) content of the studied samples.

Cultivar	Chlorophyll	Carotenoids	Alpha-Tocopherol
Nourgou	1.37 ± 0.026 ^b^	10.86 ± 0.155 ^a^	186.866 ± 1.093 ^a^
Gousalani	1.64 ± 0.017 ^a^	3.97 ± 0.045 ^b^	175.593 ± 1.011 ^b^

The results are expressed as mean value ± standard deviation (n = 3). Superscript letters within each column indicated significant differences as resulting from ANOVA and post hoc test (*p* < 0.05).

**Table 4:** Fatty acid composition (%) found in EVOO samples.

Fatty Acid/Cultivars	Gousalani	Nourgou	Significance
Myristic acid (C14:0)	0.01 ± 0.006	0.01 ± 0.001	ns
Palmitic acid (C16:0)	13.32 ± 0.29 ^b^	17.48 ± 0.50 ^a^	***
Palmitoleic acid (C16:1)	0.65 ± 0.045 ^b^	1.23 ± 0.026 ^a^	***
Margaric acid (C17:0)	0.025 ± 0.002 ^b^	0.03 ± 0.002 ^a^	ns
Margaroleic acid (C17:1)	0.04 ± 0.001 ^b^	0.05 ± 0.012 ^a^	ns
Stearic acid (C18:0)	2.65 ± 0.045 ^b^	3.52 ± 0.545 ^a^	*
Oleic acid (C18:1)	68.73 ± 0.651 ^a^	65.33 ± 0.588 ^b^	**
Linoleic acid (C18:2)	13.47 ± 0.113 ^a^	11.06 ± 0.149 ^b^	***
Linolenic acid (C18:3)	0.55 ± 0.045 ^b^	0.73 ± 0.052 ^a^	**
Arachidic acid (C20:0)	0.30 ± 0.017 ^b^	0.40 ± 0.017 ^a^	***
Gadoleic acid (C20:1)	0.20 ± 0.017 ^a^	0.11 ± 0.010 ^b^	***

Results are estimated as mean± standard deviation. ns: not significant; *p* < 0.05 *, *p* < 0.01 **, *p* < 0.001 ***; different letters indicate homogenous sub-groups from ANOVA (post hoc, *p* < 0.05).

**Table 5:** Total sterolic and total phenolics contents (TPC) detected in extra virgin olive oils.

Cultivars	Total Sterols	Total Phenolic Contents
Nourgou	1036.4 ± 147.8 ^b^	348.03 ± 2.670 ^b^
Gousalani	1931.4 ± 251.4 ^a^	516.16 ± 10.793 ^a^

Results are estimated as mean value ± standard deviation. Superscript letters (^a,b^) within each column indicate significant differences resulting from ANOVA and post hoc test (*p* < 0.05).

**Table 6:** Phenolic compound amounts (mg/kg) measured in EVOO samples.

Phenolic Class	Compound	Gousalani	Nourgou	Significance
Secoiridoids	Oleuropein	78.60 ± 0.544 ^a^	9.51 ± 0.482 ^b^	***
	Oleuropeinaglycone	164.99 ± 0.121 ^a^	1.01 ± 0.052 ^b^	***
	Ligstroside aglycone	62.040 ± 1.027 ^a^	22.46 ± 0.134 ^b^	***
	Verbascoside	4.63 ± 0.045 ^b^	7.00 ± 0.026 ^a^	***
Phenolic alcohols	Hydroxytyrosol	6.19 ± 0.190 ^a^	2.56 ± 0.088 ^b^	***
	Tyrosol	4.77 ± 0.220 ^b^	7.14 ± 0.079 ^a^	***
Phenolic acids	Caffeic	0.96 ± 0.018 ^a^	0.59 ± 0.026 ^b^	***
	Vanillic	0.74 ± 0.015 ^b^	1.46 ± 0.036 ^a^	***
	*p*-cumaric	2.10 ± 0.017 ^b^	2.53 ± 0.062 ^a^	***
	Ferulic	1.46 ± 0.036 ^a^	0.99 ± 0.020 ^b^	***
	*o*-cumaric	12.60 ± 0.017 ^a^	0.55 ± 0.045 ^b^	***
Flavonoids	Catechin	0.7367 ± 0.015	0.73 ± 0.065	ns
	Luteolin-7-*O*-glucoside	0.31 ± 0.019 ^b^	1.59 ± 0.017 ^a^	***
	Rutin	1.28 ± 0.017 ^a^	0.33 ± 0.026 ^b^	***
	Luteolin-4-*O*-glucoside	26.83 ± 0.221 ^a^	4.53 ± 0.062 ^b^	***
	Luteolin	3.66 ± 0.040 ^a^	0.78 ± 0.036 ^b^	***
	Apigenin	0.66 ± 0.045 ^b^	1.71 ± 0.078 ^a^	***
	Diosmetin	1.29 ± 0.034	1.29 ± 0.105	ns
Phenolic aldehydes	Vanillin	0.47 ± 0.030 ^b^	0.83 ± 0.034 ^a^	***
Total phenolic content		516.16 ± 14.562 ^a^	348.03 ± 2.670 ^b^	***

The data are expressed as mean values ± standard deviation. ns: not significant; *p* < 0.001 ***; different letters indicate homogenous sub-groups from ANOVA (post hoc, *p* < 0.05).

**Table 7:** Importance for prediction of the different features as related to the discrimination between the two cultivars.

VIP Features	VIP [t]	VIP [ortho-t]
*o*-coumari acid	1.502	0.025
Verbascoside	1.5015	0.048
Hydroxytyrosol	1.5013	0.083
Ligstroside aglycone	1.443	0.289
Vanillin	1.335	0.921
Total phenolic content	1.305	0.660
Caffeic acid	1.167	0.348
Oleuropein aglycone	1.127	1.220
Oleuropein	1.025	0.384
Vanillic acid	1.007	1.368

(VIP score = variable importance in projection). VIP [t] = prediction ability between-groups; VIP [ortho-t] = prediction ability within-groups.

**Table 8:** Antioxidant potential detected in olive oils analyzed using FRAP and ORAC tests.

Antioxidant Activity/Cultivar	Nourgou	Gousalani	Significance
Method 1: FRAP mg GAE/100 g	15.66 ± 6.506 ^b^	50.00 ± 9.165 ^a^	**
Method 2: ORAC mM TEAC	2024.5 ± 349.500 ^b^	8796.00 ± 343.000 ^a^	***

The data are presented as mean values ± standard deviation (n = 3); superscript letters within each column indicate significant differences according to ANOVA and post hoc test (*p* < 0.05). *p* < 0.01 **, *p* < 0.001 ***; different letters indicate homogenous sub-groups from ANOVA (post hoc, *p* < 0.05).

## Data Availability

Data are contained within the article.
